# H1 antihistamines and driving

**Published:** 2008-08-15

**Authors:** Popescu Florin-Dan

**Affiliations:** * Allergology Department, University of Medicine and Pharmacy “Carol Davila” Bucharest, Romania

**Keywords:** H1 antihistamines, sedation, cognitive and psychomotor tests, driving studies

## Abstract

Driving performances depend on cognitive, psychomotor and perception functions. The CNS adverse effects of some H1 antihistamines can alter the patient ability to drive. Data from studies using standardized objective cognitive and psychomotor tests (Choice Reaction Time, Critical Flicker Fusion, Digital Symbol Substitution Test), functional brain imaging (Positron Emission Tomography, functional Magnetic Resonance Imaging), neurophysiological studies (Multiple Sleep Latency Test, auditory and visual evoked potentials), experimental simulated driving (driving simulators) and real driving studies (the Highway Driving Test, with the evaluation of the Standard Deviation Lateral Position, and the Car Following Test, with the measurement of the Brake Reaction Time) must be discussed in order to classify a H1 antihistamine as a true non-sedating one.

**Driving** represents a complex task that integrates various mental functions in an environment that necessitates continuous adaptation, and defines the controlled operation of a land vehicle, usually a motor vehicle, such as a car or a truck. Although direct operation of a motorcycle, a bicycle or a mounted animal is usually called riding, such operators are legally considered to be drivers and are required to act according to the rules of the road. 

**Driving performances** depend on cognitive, psychomotor and perception functions. Cognitive performance is critical because driving requires the attentiveness to the driving environment, and is in constant interaction with physiological systems, especially visual information processing. Driving involves not only physical skills in order to operate the mechanisms which control the vehicle, but also mental skills, including those required to apply the rules of the road. In some countries, physicians evaluate patients whom they identified as suffering from a deterioration of skills that could result in dangerous or incompetent driving, using cognitive and psychomotor psychometrically tested tools (Weiss and Ratzon, 2007).

**Driving accidents** are road traffic incidents which usually involves at least one road vehicle being in collision with another vehicle, another road user, or a stationary roadside object, and which usually results in injury or property damage. Car accidents result in the deaths of over one million people worldwide each year. Ability to drive safely is the resultant of interactions between the individual (the driver), the vector (the car) and the environment (the state of the road) (Gonthier et al, 2005). 

The main **causes of traffic accidents** may be grouped into the following scenarios: fatigue, sleepiness or distraction of the driver, alcohol and/or drugs and/or diseases that alter the proper ability to drive, loss of control due to slippery road, inexperience, excessive speed or disobedience of traffic safety rules, and diverted attention, mainly by mobile phones or music players. 

Many car crashes involve more than one contributing factor, including speed, loss of control, and slippery roads (Braitman et al, 2008). Automobile crash reports show that a great part of fatal crashes involve alcohol. Laboratory studies show that alcohol increases impulsive behaviors by impairing the drinker's ability to inhibit inappropriate actions and this effect can be exacerbated in conflict situations (Fillmore et al, 2008). Both females and subjects with lower body mass index are at an increased risk of exceeding the legal limit of breath alcohol concentration after moderate alcohol consumption resembling a social drinking (Barquin et al, 2008). Efforts to prevent accidents include prevention of alcohol use and treatment of alcohol dependence (Hingson et al, 2008). In-vehicle distraction (entertainment systems and telephones) are reported as a contributory factor in some of the fatal accidents (Stevens and Minton, 2001). Moreover, there is a frequent disparity in real life between awareness regarding the mandatory use of seatbelts and actual use (Okpala et al, 2007). 

Excessive daytime sleepiness or **somnolence** is a common symptom, with a prevalence of 10–20% in the general population (Carskadon, 1993). The risk of car accidents is assessed in many medical conditions based upon sleepiness severity. In a study performed on more then three thousand persons in order to evaluate the use of sleepiness countermeasures among drivers, four clusters of behavior were identified: alertness-enhancing activity while driving (turn on the radio/CD, ask passengers to engage in conversation), stopping the car, taking a nap and ingesting coffee or other sources of caffeine, such as energy drinks (Anund et al, 2008). 

**Untreated allergy symptoms**, such as sneezing in allergic rhinitis or pruritus in urticaria, can distract attention in patients driving. Clinical manifestations of nasal allergy may cause sedation and altering of cognitive functions. Ocular itching and increased lacrimation in allergic conjunctivitis can affect the proper use of visual capacities.

**Analytical studies** (case-control or retrospective cohort surveys) have been used to correlate an increased risk of accident among people who use a certain type of drug. Psychoactive drugs, sedatives, benzodiazepines, minor and major tranquilizers, tricyclic antidepressants and opiate analgesics are cited to have a significant relative risk (Jauregui et al, 2006). Although the observed relative risk for H1 antihistamines appears to be generally low in few such studies, first-generation H1 antihistamines are sedating and pose potential risks for those operating machinery or driving a car. There are many OTC (Over The Counter) drugs for the treatment of coughs and colds, allergies, pain, nausea and gastrointestinal upsets, with the potential to cause sedation, first generation H1 antihistamines being recorded as having the greatest sedative effect (Department of Transport, 2000). These first generation drugs cause excessive dizziness and sedation in older persons. Alcohol intensify the sedation caused by older H1 antihistamines (Dufour et al, 1992).

**Sedation** is the depression of brain functioning by a medication manifested not only by sleepiness, drowsiness and reduced wakefulness, but also by impaired cognitive and psychomotor performances. The depressant effects of a evening dose of a first generation H1 antihistamine persist through the next day. People may fail to recognize or may underestimate the adverse effects on the CNS resulting from the use of H1 antihistamines (Kay, 2000). 

The CNS depressive effects of the first generation H1 antihistamines cause drowsiness, fatigue, somnolence, dizziness, impairment of cognitive functions, reduced psychomotor performance, with incoordination, and increased reaction time. Moreover, in many cases they also induce peripheral neurological actions secondary to *anticholinergic effects* (dilatation of the pupils, blurry vision, or dry mouth), which can affect patient ability to drive (Jauregui et al, 2006).

H1 antihistamines are antiallergic drugs that act on H1 receptors (H1Rs), and because there are no differences between the CNS and peripheral organ H1R (Timmerman, 2000; Schwartz and Arrang, 2002), their central nervous system (CNS) adverse effects depend on the penetration of the blood-brain barrier and the degree of H1R occupancy in the human brain (Popescu et al, 2007a), usually evaluated using positron emission tomography (PET) (Tashiro et al, 2006). 

Neural histamine and H1Rs are involved in the sleep-wake mechanism. Center of wakefulness histaminergic neurons in the posterior hypothalamus induce arousal by H1-mediated depolarization of thalamic relay neurons, excitation of neocortical pyramidal neurons, and excitation of ascending cholinergic neurons (Schwartz and Arrang, 2002; Yanai and Tashiro, 2007). 

H1Rs are involved in cognitive processes, such as learning and memory, for which the frontal cortex, amygdala and hippocampus interact (Dai et al, 2007). In normal human subjects, the brain density of H1Rs is high in the medial and dorsolateral prefrontal, anterior cyngulate, temporal, occipital cortices, thalamus, amygdala and hippocampus (Yanai and Tashiro, 2007).

Factors that define the criteria for H1 antihistamines to be non-sedative are: incidence of sleepiness, alterations of cognitive and psychomotor functions, but also measurements of CNS H1-receptor occupancy (H1RO) profile using PET (Holgate et al, 2003). 

**A new classification of H1 antihistamines** according to their PET H1R occupancy in the human brain is emerging. Oral H1 antihistamines can be *non-sedating *(H1RO less than 20%), such as fexofenadine (120 mg), epinastine (20 mg), ebastine (10 mg), cetirizine (10 mg) and olopatadine (5 mg); *less-sedative* (H1RO of 20-50%), such as azelastine (1 mg), mequitazine (3 mg) and cetirizine (20 mg), and *sedative* (H1RO more than 50%), such as first generation H1 antihistamines d-chlorpheniramine (2 mg), oxatomide (30 mg) and ketotifen (1 mg) (Yanai and Tashiro, 2007).

Besides PET and functional Magnetic Resonance Imaging (fMRI), the **physiological evaluation of sedation** includes the *Multiple Sleep Latency Test* (MSLT) and *evoked potentials*. 

The use of MSLT reveal that the sedative effects of a first generation H1 antihistamine, such as chlorpheniramine, administered in the evening persist through the next day (Starbuck et al, 2000). *Auditory evoked potentials* (AEP), measuring the P300 event-related potential (P300) latency parameter, were used to evaluate the CNS effects of H1 antihistamines in several studies. Cetirizine and loratadine, in doses of 10 mg p.o., do not induce a significant increase in the mean P300 latency, while diphenhydramine 50 mg increases it (Simons et al, 1996a, 1996b; Simons FE, 1999). 

In two placebo-controlled studies, we used *Visual Evoked Potentials* (VEP) recordings in order to provide important information regarding the effects of second generation H1 antihistamines on the functional integrity of the visual system. Loratadine 10 mg p.o. or levocetirizine 5 mg p.o. induced no statistically significant changes, neither in the mean values of all P100 parameters, nor in the mean latency of N75 and N135 components. No unilateral or bilateral alterations of VEP parameters were detected, suggesting that neither loratadine nor levocetirizine had any effect on the functional integrity of visual sensorial pathway. Our studies initiated a novel approach in the neurological safety profile evaluation of new H1 antihistamines using VEP, with the advantage of no radiological exposure (Popescu, 2005; Popescu et al, 2007b). 

**Standardized cognitive and psychomotor tests** for evaluation of sedation include *Choice Reaction Time* (CRT), *Critical Flicker Fusion *(CFF), and *Digital Symbol Substitution* (DSST) tests. In the CRT test subjects are required to extinguish one of several lights, illuminated at random, by pressing a button in front of the light. Hydroxyzine 50 mg orally causes significant impairment in RRT (*Recognition Reaction Time*) and TRT (*Total Reaction Time*) versus placebo, alone and moreover with alcohol (0.3 g/kg body weight or approximately 0.43-0.50 g/L blood-alcohol concentration), while fexofenadine 180 mg p.o. with or without alcohol has no significant effect on CRT parameters (Ridout et al, 2003). 

In the CFF evaluation subjects define the frequency required for a sequence of blinking lights to remain fixed or continuous. Levocetirizine 5 mg p.o. does not modify CFF when compared with placebo, in contrast with diphenhydramine 50 mg (Gandon and Allain, 2002). DSST involves the substitution of simple figures/symbols for digits. The number of correct symbols substituted for digits during a time-limited period is measured. Fexofenadine, in a high dose of 360 mg p.o., does not affect cognitive performance measured by DSST (Theunissen et al, 2006). 

Using such objective tests it was proven that first-generation H1 antihistamines can be clearly related to car accidents. Traffic accidents are six times more likely if the driver was taken sedating H1 antihistamines, while the use of cellular phones in motor vehicles is associated with a quadrupling of the risk of a collision during the brief time interval involving a call (Redelmeier and Tibshirani, 1997; Hindmarch and Shamsi, 2001).

Small body mass and the use of large doses of first generation H1-antihistamines, hepatic or renal dysfunctions leading to increased accumulation in the CNS, and the concomitant use of other drugs or alcohol that impair the CNS function may amplify the risk of adverse effects involved in car accidents. Clinical tolerance to the sedating effects of these drugs was been reported anecdotally, but it has not consistently been found objectively (Simons, 2004).

**Determination of drugs in biological fluids** of traffic accident victims (deaths or injuries) documented a prevalence of antihistamines of 0.6% (Jauregui et al, 2006). 

In some countries, about 5% of drivers may use an antihistamine before driving. The sedative effects of first generation H1 antihistamines may be fatal among car drivers. In an evaluation of the incidence of drug use among individuals killed in road traffic accidents, sedative H1 antihistamines were found to be responsible for 72% of deaths compared with 87% for alcohol, 90% for cannabinoids, and 97% for tranquillisers and antidepressants (Hindmarch, 2002). 

**Experimental simulated driving studies** use driving simulators as a complement to psychomotor testing, in order to evaluate the true effect of H1 antihistamines in safer conditions than under real driving condition (on-street driving). The Iowa Driving Simulator consists of a domed enclosure mounted on a hexapod motion platform, with the inner walls of the dome acting as a screen on which correlated images are projected. The experimental drive (conducted in dry weather conditions, good visibility, low-density traffic, and with a posted speed limit) has two phases, driven consecutively without interruption, in the first one the driver follows a car, and in the second one he continues to drive normally along the designated route. In a study using this simulator, subjects had significantly better coherence after ingesting alcohol, approximately 0.1% blood alcohol concentration (21.7 mmol per liter), or fexofenadine 60 mg p.o. than after taking diphenhydramine 50 mg. Lane keeping was impaired (steering instability and crossing the center line) after alcohol and diphenhydramine use compared with fexofenadine (Weiler et al, 2000). 

**Experimental real driving** studies are performed using the *highway driving test and the car following test*. 

Experimental **real driving tests on the highway** at a constant speed (90-95 km/h), in a 100 km circuit (50 km either way) and a stable position in the right lane, are carried out during normal traffic, in adapted automobiles with infrared distance sensors, camera, duplicated controls, a computer to monitor speed, angle of turn and deviation from midline, and EEG and ECG driver monitoring. 

*Standard Deviation Lateral Position* (SDLP) parameter is measured in the protocolized driving test, and represents an index of road tracking error or ‘weaning” reflecting the driver’s ability to keep the vehicle in the center of the lane. SDLP is a reliable characteristic of individual driving performance, sensitive to many sedating agents, including alcohol and H1 antihistamines (Jauregui et al, 2006).

**First generation H1 antihistamines** significantly affect driving ability, both after single dosing and in the context of repeated daily dosing (**Table I**). Sedating H1 antihistamines have comparable or greater impact on driving ability than alcohol. Triprolidine 10 mg is equivalent to the effect of blood alcohol level of 0.05 mg/dL (SDLP +2.6 cm), effect lasting up to four hours (O’Hanlon et al, 1988).

**Table 1:** **First generation H1 antihistamines in real driving conditions** (modified from Jauregui et al, 2006)

Drug	Dose	Sex	Day 1*	Day 2**
Clemastine	4 mg/day	M, F	SD	SD
Diphenhydramine	50 mg/day	M, F	SD	SD
Dexchlorfeniramine	6 mg/day	M, F	SD	NT
Triprolidine	10 mg/day	M	SD	SD
	10 mg/day	M,F	SD	SD
*single dose,**daily dose, M = males, F = females, SD = significant differences vs placebo, NT = not tested				

Many second generation H1 antihistamines, such as **loratadine, fexofenadine and levocetirizine**, do not affect driving performance (**Table II**). 

**Fexofenadine** is nonsedating and does not impair performance or driving ability, even at high doses (Meeves and Appajosyula, 2003). Driving performance is not significantly affected while using 5 mg **levocetirizine** once daily (Verster et al, 2003).

Certain H1 antihistamines with alcohol have potential additive effects in impairment of the ability to drive. Cetirizine causes impairment of driving performance after a single 10 mg p.o. dose when associated with alcohol (0.72 g/kg, lean body mass) (Ramaekers et al, 1992). 

**Desloratadine** 7.5 mg p.o. does not potentiate alcohol-mediated CNS impairment (alcohol weight adjusted to an average blood alcohol concentration of 0.1%). With or without alcohol, desloratadine does not increase sedation or impair psychomotor performance (Scharf M, Berkowitz D, 2007).

Combination of second generation H1 antihistamines with ***pseudoefedrine*** does not improve the ability to drive versus the antihistamine alone (Jauregui et al, 2006). 

Many **second-generation H1 antagonists** can be safely co-administered with alcohol, sedatives, hypnotics, antidepressants, or other CNS-active substances (Simons, 1999), but important precautions are imposed when the patient is driving.

**Table 2:** **Several second generation H1 antihistamines in real driving conditions** (modified from Jauregui et al, 2006)

Drug	Dose	Sex	Day 1*	Day 2**
Loratadine	10 mg/day	M, F	NS	NS
Fexofenadine	120-240 mg	M, F	NS	NS
Levocetririzine	5 mg/day	M, F	NS	NS
*single dose,**daily dose, M = males, F = females, SD = significant differences vs placebo				

Another new generation H1 antihistamine, **desloratadine**, does not impair the driving performance. In a randomized, double-blind, crossover study, there were no significant differences between desloratadine 5 mg p.o. and placebo in SDLP, whereas diphenhydramine 50 mg significantly increased this parameter. Moreover, desloratadine and fexofenadine have the abbility to return allergic patient's performance back to normal (Theunissen et al, 2006). 

In a **car following test**, the test battery includes critical flicker fusion, *Choice Reaction Time and Brake Reaction Time* (BRT), in order to evaluate the cognitive and psychomotor performance to respond by depressing the foot pedal to random red braking lights of a vehicle ahead simulated with lights mounted on the hood (BRT). The automatic car was driven with a speed of 40 km/h, on a close driving course (2 straight lanes 1.5 km connected by U shaped turning roads on ends). 

Hydroxyzine 50 mg p.o. cause impairment in BRT, but **fexofenadine** 180 mg p.o., alone or with alcohol (0.3 g/kg body weight or approximately 0.43-0.50 g/L blood-alcohol concentration) has no significant effect on this parameter (Ridout et al, 2003).

BRT was significantly faster in a study following treatment with **desloratadine** than diphenhydramine or placebo (Vuurman et al, 2004).

There are several **limitations for the studies** under real driving conditions: interindividual variability, studies in healthy volunteers *versus* patients with allergic diseases, variability of blood drug concentrations, and variability according to gender. Women seem to be more sensitive to the effects of some H1 antihistamines, such as acrivastine or emedastine, but there are no differences between males and females for fexofenadine and levocetirizine (Jauregui et al, 2006).

Loratadine, desloratadine and fexofenadine are oral second generation H1-antihistamines that may be used by licensed pilots (Popescu, 2007c), after adequate individual experience has determined that these drugs are well tolerated without significant side effects.

In **conclusion**, first generation H1 antihistamines, such as diphenhydramine, triprolidine, hydroxyzine, clemastine or dexchlorpheniramine, significantly impair driving performance after both single dose or repeated administration. Some second generation H1 antihistamines, such as cetirizine, may also impair driving performance, but the magnitude and extent of impairment depend on the administered dose. New H1 antihistamines, such as fexofenadine, desloratadine and levocetirizine, produce no driving impairment (Vuurman et al, 2004; Verster and Volkerts, 2005).
